# Telitacicept in combination with B-cell depletion therapy in MuSK antibody-positive myasthenia gravis: a case report and literature review

**DOI:** 10.3389/fimmu.2024.1456822

**Published:** 2024-11-18

**Authors:** Jia Wang, Haotao Zheng, Jia Wei, Jiaping Wu, Ziyang Feng, Xueqin Chen, Yangsicheng Liu, Wenxin Qin, Xiude Qin, Fanxin Kong

**Affiliations:** ^1^ The Fourth Clinical Medical College, Guangzhou University of Chinese Medicine, Shenzhen, China; ^2^ Encephalopathy and Psychology Department, Shenzhen Traditional Chinese Medicine Hospital, Shenzhen, China; ^3^ Acupuncture and Moxibustion Department, Shenzhen Traditional Chinese Medicine Hospital, Shenzhen, China

**Keywords:** myasthenia gravis, muscle-specific tyrosine kinase, telitacicept, B-cell depletion therapy, case report

## Abstract

Muscle-specific kinase (MuSK) myasthenia gravis (MG) is relatively rare and has a higher incidence of myasthenic crisis compared with other subtypes. However, there is still a lack of effective treatment for refractory MuSK MG. We report the case of a 70-year-old female MuSK MG patient with recurrent fluctuations who stabilized on telitacicept in combination with anti-CD20 B-cell depletion therapy. This combination regimen deserves further investigation. Furthermore, we summarized the treatment protocols of 14 previously reported cases of MuSK MG.

## 1 Introduction

Myasthenia gravis (MG) is an autoantibody-mediated autoimmune neuromuscular junction (NMJ) disorder that impairs neuromuscular excitatory transmission and is clinically characterized by fluctuating muscle weakness and fatigue (1). MG is associated with antibodies directed against the acetylcholine receptor (AChR), muscle-specific kinase (MuSK), lipoprotein-related protein 4 (LRP4), titin and agrin in the postsynaptic membrane at the neuromuscular junction (2). Approximately 5% of patients with MG previously diagnosed as negative for anti-acetylcholine receptor (AChR) antibodies (AChR-Abs) are found to be positive for muscle-specific tyrosine kinase antibodies (MuSK-Abs) (3). Its prevalence in patients with myasthenia gravis is roughly between 20% ~ 30%, especially in MuSK MG, where the bulbar and respiratory muscles are preferentially involved and the incidence of myasthenia crisis and respiratory failure is much higher (4). B-cell depletion therapy (BCDT) refers to a treatment that consumes, removes, or inhibits B cells through a certain pathway during the development, maturation, activation, and differentiation of B cells (5). Telitacicept (China, RC-18) is a novel, recombinant fusion protein, consisting of transmembrane activator and calcium modulator and cyclophilin ligand interactor (TACI) and the Fc portion of human immunoglobulin G (IgG) (TACI-Fc). It was designed to inhibit the activity of two target cytokines, the B-cell lymphocyte stimulator (BLyS, also known as the B-cell activation factor [BAFF]) and a proliferation-inducing ligand (APRIL), both of which are involved in B cell-mediated autoimmune diseases (6). Its phase 3 clinical trial for the treatment of MG is ongoing, but there are few cases of MuSK MG treated with telitacicept. Herein, we report a patient with refractory MuSK MG who showed benefit from treatment with telitacicept. However, her symptoms were prone to relapse and the disease was stabilized in combination with anti-CD20 B-cell depletion therapy.

## 2 Case description

A 70-year-old female patient was admitted to hospital in April 2023. She presented with generalized weakness, slurred speech, and dizziness. Serology showed negative for AChR-Ab and positive for MuSK-Ab [1:40, as determined by cell-based assays (CBA)]. Chest computed tomography (CT) and whole-body positron emission tomography-computed tomography (PET-CT) were performed to rule out the possibility of thymoma tumors. She was definitively diagnosed with MG and started on prednisone and pyridostigmine with poor results and severe gastrointestinal reactions to tacrolimus. The disease progressively deteriorated and the patient developed MC, which was controlled and relieved after PLEX. However, the patient failed to adhere to oral medications, resulting in a relapse of symptoms after two weeks, and was admitted to our hospital in June 2023. Her clinical manifestations include difficulty lifting the neck, dysphagia, dysarthria, fluctuating ptosis, and limb weakness. On the day of admission, she completed a neurological examination and was tested with a quantitative MG (QMG) score of 18 points and MG-specific activities of daily living scale (MG-ADL) score of 10 points (Myasthenia Gravis Foundation of America IIIb, MGFA IIIb), and serological immune testing for MuSK-Ab (1:320, tested by CBA) (**Supplementary Figure 1**).

## 3 Therapeutic intervention and follow-up outcomes

The patient was treated with mycophenolate mofetil (MMF) (1 g/day) and pyridostigmine (90 mg/d). However, pyridostigmine caused gastrointestinal discomfort and symptoms did not relieve. One week after admission, she experienced dyspnea and a drop in oxygen partial pressure, which indicated MC. She started treatment with PLEX (once every 2 days) and high-dose intravenous methylprednisolone (1,000 mg daily for 3 days, gradually reduced to 240 mg/day). MMF was maintained at the same level while pyridostigmine was discontinued and her limb weakness and dyspnea improved after 5 PLEX. Since the middle of July 2023, she started treatment of telitacicept (160 mg/week, total of eight injections). After each injection, there will be scattered red rashes, swelling, and slight pain at the injection site, but the patient can tolerate it. Two weeks after the initial injection, a significant clinical improvement was observed (QMG score of 14 and MG-ADL score of 7). After eight injections, her symptoms of muscle weakness and dyspnea have improved well, and her and dysphagia is better than before (QMG and MG-ADL decreased by 8 points and 5 points, respectively, from baseline).

Since January 2024, she had frequent exacerbations or MC. She developed generalized pain and headache after receiving efgartigimod injection at another hospital, and no beneficial effect was observed with IVIG. Due to unsatisfactory results, the patient returned to our center. The patient’s symptoms improved with PLEX and telitacicept, and the MuSK-Ab level had reduced to 1:100 in April 2024. However, the condition still fluctuated. For the recurrent and refractory nature of the patient’s condition, we considered it to be related to the difficulty in complete clearance of the B-cells and used rituximab (375 mg/m^2^/6 months) in combination with telitacicept in late March 2024. One month later, the patient was admitted to hospital with herpes zoster virus infection, with worsening limb weakness but no dysphagia or ptosis and clear speech (QMG score of 16, MG-ADL score of 2), which improved after antiviral therapy. After 3 months of follow-up, the patient’s symptoms continued to improve (QMG score of 12, MG-ADL score of 2). The prednisone dose was progressively reduced from 30 to 10 mg per day, and MMF was reduced to 0.5g per day.

## 4 Literature review

A literature review of similar case reports was conducted using PubMed, and 14 MuSK MG-associated case reports were identified after excluding cases with coexisting infectious diseases (**Table 1**). Of the 14 patients in this review, 8 were positive for MuSK antibodies and 6 were positive for MuSK in combination with other subtypes, 7 individuals (50%) had a history of MC. All patients who underwent PLEX eventually showed benefits, especially those who experienced MC, but there was no absolute benefit from IVIG, with only 2 of the 6 cases using IVIG showing improvement in symptoms as a result, cholinesterase inhibitors also responded poorly in MuSK MG, these are consistent with previous studies (7). Of the 16 documented patients, 6 used the biologic agent, all rituximab. Of these, 5 showed eventual benefit, 2 showed IgE-mediated or direct mast cell activation reactions (cases 9 and 10), and because there was no reasonable alternative treatment regimen, the patients’ symptoms eventually improved after insistence on desensitization. Patients with a combination of MuSK and other types of antibodies responded well to treatment with pyridostigmine (cases 2, 3, 4 and 7), which appears to be related to the presence of AChR antibodies and the relatively low titer of antibodies to MuSK. Glucocorticoids or corticosteroids were used in all reported cases, but their role in MuSK MG is difficult to assess, and in some cases, symptoms can be improved by using low-dose corticosteroids in the initial treatment, while in some refractory cases, a mega dose of methylprednisolone in combination with IVIG not result in a complete clinical benefit (cases 9, 11 and 12). For patients who have experienced MC, symptoms are more likely to fluctuate and worsen again, and the effectiveness of a treatment regimen needs to be assessed with longer follow-up.

**Table 1:** Literature review of patients with MuSK-MG.

Patients	Study, year	Race	Sex/Age(y)	Subtype	Antibody Testing	MC	Primary treatments	Biological agents	Treatments of ultimate benefit	Response to treatment
1	Carolyn Tsai, 2021 (22)	African American	F/40	MuSK-ab	MuSK antibody—60.7 nmol/L n.v.<0.02)	Yes	pyridostigmine, prednision, PLEX	RTX	PLEX, prednision, RTX	improvement to reliance on BiPAP at night; MuSK antibody—0.15 nmol/L
2	C. Rodolico, 2023 (23)	European	M/86	AChR-ab + MuSK-ab	AChR antibody—10.72 nmol/l (n.v. < 0.2); MuSK antibody—1.7 nmol/ml (n.v. < 0.05; radioimmunoassay method)	No	Pyridostigmine prednisone	No	Pyridostigmine, prednisone	AChR Abs remained positive (3.78 nmol/l), but MuSK Abs became negative (0.007 nmol/ml)
3	Weibin Liu, 2020 (24)	Asia	F/12	AChR-ab + MuSK-ab	AChR antibody—0.56 nmol/L (n.v. <0.45) and MuSK antibody—1.15 nmol/L (n.v. < 0.05)	Yes	Pyridostigmine, methylprednisolone, azathioprine	No	Pyridostigmine, methylprednisolone, tacrolimus	MuSK-Ab (1.44 nmol/L); AChR-Ab was not detected
4	Weibin Liu, 2020 (24)	Asia	M/7	AChR-ab returned to AChR-ab + MuSK-ab	AChR-Ab was positive (1.4 nmol/L) and MuSK-Ab was negative	No	Pyridostigmine, IVIG	No	Pyridostigmine, Prednisone	AChR-Ab (1.46 nmol/L) and MuSK-Ab (0.08 nmol/L)
5	S.A. Patil, 2018 (25)	Asia	M/32	AChR-ab + MuSK-ab + LRP4-ab	The cut-off value of AChR, MuSK and LRP4 ELISA were 0.568, 0.751 and 0.621 respectively	No	Pyridostigmine	No	PLEX	Improved; No deterioration in condition within one year
6	Koji Abe, 2018 (26)	Asia	M/37	MuSK-ab	MuSK antibody level at 6.03 nM (cut-off value=0.05 nM)	No	Tacrolimus, IVIG, prednisolone, cyclosporine	No	Alternative PLEX and double-filtration plasmapheresis	The variation in the anti-MuSK antibody level almost disappeared
7	Tatsusada Okuno, 2021 (27)	Asia	F/62	MuSK-ab + Titin-ab + LRP4-ab	MuSK antibody — 28.6 nmol/L (n.v. <0.02; RIA). Anti LRP4 Ab measured by luciferase immunoprecipitation systems was 62,568 relative light units (RLU) (positive control, 58,682 RLU). Titin antibody 1.18 (n.v. < 1.0; CBA)	Yes	Tacrolimus	No	Methylprednisolone, pyridostigmine, cyclosporine	Improved
8	Steven Weger,2019 (28)	America	F/6	MuSK-ab	MuSK antibody— 1:160 (normal <1:10)	Yes	Pyridostigmine, methylprednisone	RTX	IVIG, RTX	Improved
9	Joome Suh, 2019 (29)	America	F/29	MuSK-ab	NM	No	Pyridostigmine, immunoglobulins, methylprednisone, MMF	RTX	RTX	Improved
10	Joome Suh, 2019 (29)	America	F/35	MuSK-ab	NM	Yes	Pyridostigmine, prednisone, MMF	RTX	RTX	Improved
11	Can Ebru Bekircan-Kurt, 2019 (30)	Asia	F/14	MuSK-ab	MuSK antibody— 2,15 nmol/L (Normal: < 0.05 mol/L; RIA)	Yes	Pyridostigmine, IVIG, cyclosporine	RTX	PLEX, MMF	Improved
12	Can Ebru Bekircan-Kurt, 2019 (30)	Asia	NM/NM	MuSK-ab	MuSK antibody— 2,12 nmol/L (Normal: < 0.05 mol/L; RIA)	No	Pyridostigmine, methylprednisone, IVIG	No	Cyclosporine	Improved
13	T.J.S. Bekooij, 2020 (31)	European	M/58	MuSK-ab	MuSK antibody— 20 (reference value <1)	Yes	Prednisolone, IVIG, PLEX, azathioprine	No	Prednisolone, IVIG, PLEX, azathioprine	Improved
14	Soumya Sundaram 2022 (32)	Asia	F/37	AChR-ab + MuSK-ab	The titers of MuSK and AChR Abs were 21.5nM (positive > 0.03) and 1nM (positive > 0.6), respectively.	No	Prednisolone	RTX	Prednisolone, RTX	The titers of MuSK and AChR Abs were 7nM and 0.35nM, respectively.

AChR-Ab, anti-acetylcholine receptor antibody; CBA, cell-based assays; F, female; IVIG, intravenous immunoglobulin; MuSK-Ab, muscle-specific tyrosine kinase; M, male; MC, Myasthenic crisis; MMF, mycophenolate mofetil; NM, not mentioned; PLEX, plasma exchange; RIA, radioimmunoassay; RTX, Rituximab; Y, years.

## 5 Discussion

Traditional treatments for MG encompass cholinesterase inhibitors such as pyridostigmine, thymectomy, and immunosuppressive agents, IVIG and PLEX are standard in managing MC (2). However, MuSK MG patients typically exhibit minimal thymic pathology, demonstrate an absence of response to thymectomy (2). Additionally, the antibodies in AChR-positive MG belong to the IgG1 and IgG3 subclasses, while most pathogenic antibodies of MuSK-Abs are of the IgG4 subclass, which can neither activate complement nor induce antigenic modulation (8), and only weakly bind Fc receptors expressed on immune cells, masking the site of normal MuSK-LRP4 interaction, thereby impeding AChR aggregation and impairs their alignment in the postsynaptic membrane (7, 9). Even with increased acetylcholine (as provided by pyridostigmine), the AChRs are not properly organized or functional. Reduction of IgG autoantibodies is therefore a possible therapeutic target for the treatment of generalized MuSK MG (10). The principle of action of FcRn, the neonatal Fc receptor, is that it binds to the Fc region and rescues IgG from lysosomal acidic degradation, thus promoting recycling (9). BlyS, alternatively called B-cell activating factor (BAFF), and APRIL are trimeric members of the tumor necrosis factor (TNF) family, play crucial roles in promoting the survival, proliferation, and differentiation of B cells to enhance immune responses. BLyS exerts its effect by binding to three receptors on the B cells surface: BAFF receptor (BAFF-R), B cell maturation antigen (BCMA) and transmembrane activator and cyclophilin interactor (TACI). In contrast, APRIL binds to BCMA and TACI. BAFF-R regulates immature B cell development and maturation, TACI oversees mature B cell differentiation, and BCMA promotes plasma cell survival and antibody secretion (11). Telitacicept is a novel fully human TACI-Fc fusion protein, by blocking BlyS and inhibiting APRIL, hinders the further maturation of immature B cells and the differentiation of mature B cells into plasma cells. This interference affects the secretion of autoreactive plasma cells autoantibodies and may curtail the survival of pathogenic short-lived plasmablasts cells, thereby exerting better control over disease activity by reducing serum BLys levels. Clinical studies in China are underway to explore telitacicept’s efficacy in neuroimmune diseases, including multiple sclerosis and neuromyelitis optica spectrum disease (12). MuSK MG patients exhibit elevated levels of BLys, a potential key factor in the generation, maturation and survival in autoreactive B cells in MuSK MG. It is currently hypothesized that short-lived plasmablasts play a crucial role as autoantibody producers in MuSK-MG (13). A phase 2 clinical trial enrolled AChR-Ab positive patients for MG treatment with telitacicept, showing clinically significant efficacy with a mean reduction in QMG score from baseline to week 24 was 7.7 and 9.6 in the 160 mg and 240 mg groups, respectively (14). However, data on telitacicept’s treatment of MuSK-Ab seropositive MG are currently lacking. In addition, a retrospective study assessed the effectiveness of telitacicept in patients with refractory gMG. The results demonstrated a majority of patients experienced symptomatic improvement within the initial 3 months, and this improvement was maintained at 6 months, which demonstrates prolonged pharmacodynamic formation and elimination of telitacicept (15).

Circulating short-lived plasmablasts and bone marrow–inhabiting plasma cells may contribute to MuSK MG autoantibody production (16). The anti-CD20 B cell depletion therapy (BCDT) therapeutic benefit in patients with MG. International guidelines recommend that rituximab (RTX) should be considered as an early therapeutic option in patients with MuSK MG who have an unsatisfactory response to initial immunotherapy (17). Previous reviews have also summarized various studies on RTX, and the results have shown that patients with refractory MG responded well to RTX treatment (9, 18). However, BCDT eliminates CD20+ memory and naive B cells but does not directly eliminate plasmablasts or plasma cells, and a proportion of B cell clones persist through treatment (16). The clinically proven efficacy of BCDT is not standing, some MuSK MG patients experience relapse after an initial remission (19). The mechanisms may be that most plasma cells do not express CD20, but can produce a portion of circulating Ig (13), or it could be due to the higher BAFF levels, which promote long-term survival of short-lived plasma cells (20). Thus, even when BCDT controls the disease, has limited impact on these cells and on antibody levels (5). In contrast to RTX, telitacicept can inhibit the survival of long-lived plasma cells developed into potential autoantibody-producing cells, and to avoid increased BLyS levels following RTX treatment, using telitacicept as BLyS/APRIL targeted drugs may be an avenue to improve BCDT and improve safety and efficacy of RTX (6).

**Figure 1:**
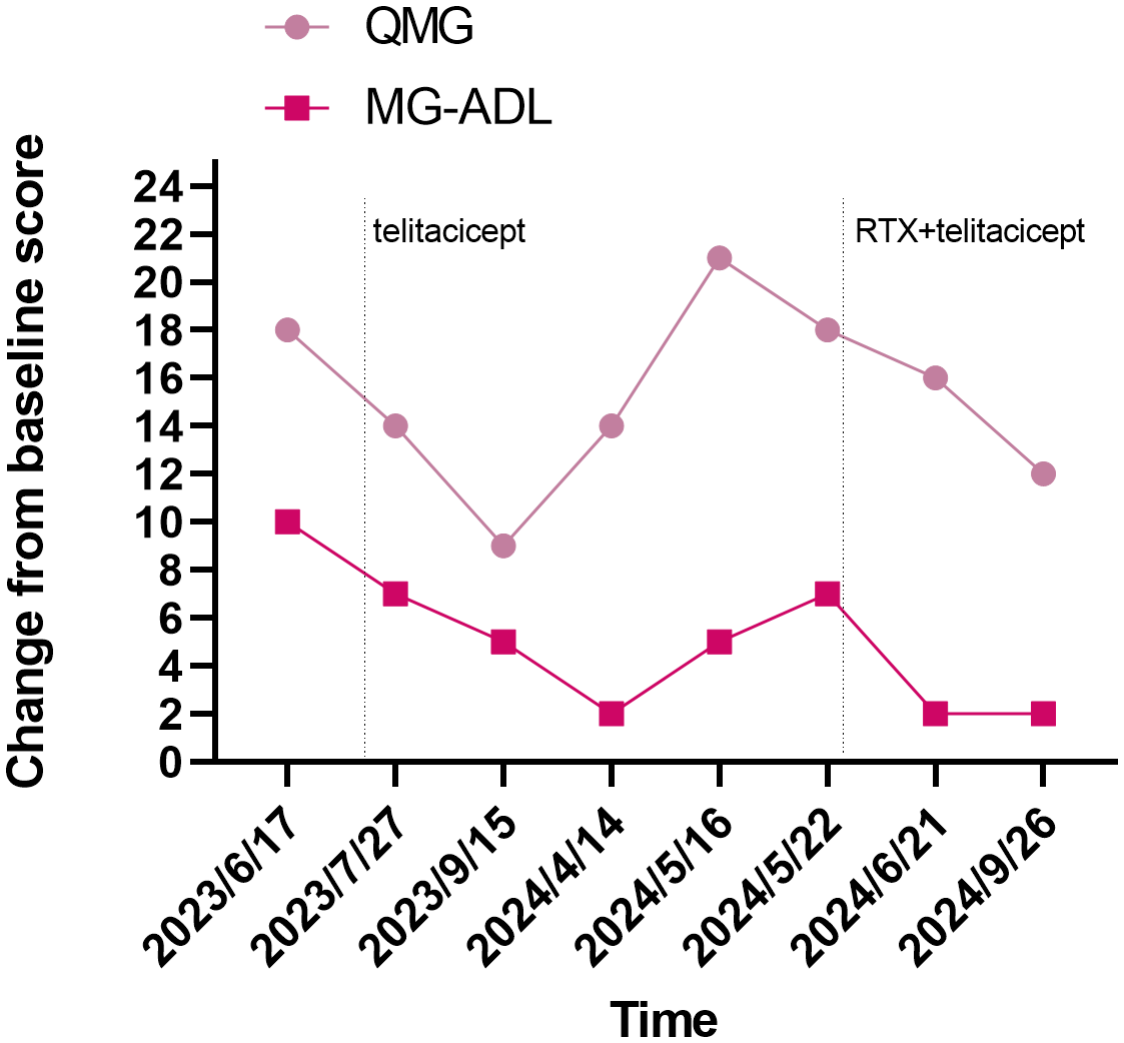
Evolution of clinical severity of MG in the patient, assessed through QMG score and MG-ADL score. MG-ADL, myasthenia gravis specific activities of daily living scale; QMG, quantitative myasthenia gravis score.

**Figure 2:**
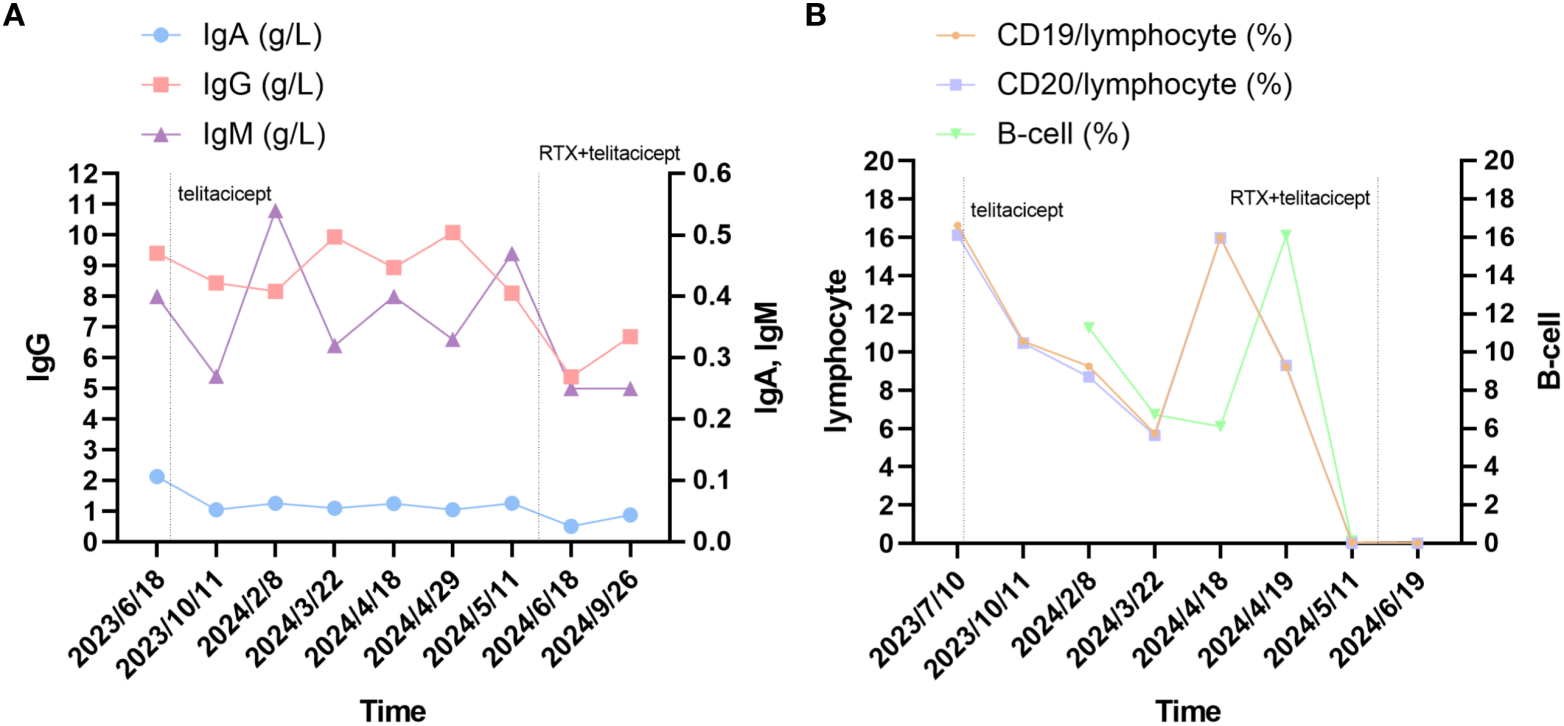
Changes in serum immune markers in the patient. **(A)** changes in levels of serum IgA, IgG and IgM. **(B)** The changes in lymphocyte and B-cell. Laboratory reference range for Indicators: IgG: 8.60-17.40 g/L; IgA: 1.00-4.20 g/L; IgM: 0.50-2.80 g/L; CD19/lymphocyte: 5-22%; CD20/lymphocyte: 5-22%; B-cell: 5-18%.

In our case report, the conventional therapeutic effect is not satisfactory, no benefit was observed with IVIG, and the patient had limited treatment options. PLEX rapidly controlled the disease when she was deteriorating or in MC, and telitacicept induced further symptomatic relief. The patient’s QMG and MG-ADL scores decreased, and laboratory tests including B cells, immunoglobulins, and lymphocytes levels showed a downward trend. There was also a decline in serum titers of MuSK-ab. Owing to the patient’s symptoms still fluctuated. We tried RTX in combination with telitacicept, and the results were surprising. Her laboratory tests levels continued to decrease, indicating that pathogenic antibodies were suppressed. Except for an episode of herpes zoster virus infection, her condition continues to improve. The detailed changes in scores are presented in **Figure 1** and changes in levels of serum IgA, IgG and IgM, and lymphocyte immunity are presented in **Figures 2A, B**.

As a specific immunosuppressive treatment, PLEX used in MC or maintenance therapy for patients with refractory MG (21). The treatment effect is usually restricted to 2–3 months, owing to continuing pathogenic antibody synthesis (2). Therefore, the potential synergistic effect of PLEX in the therapeutic phase of telitacicept cannot be ignored. In particular, no scores were recorded prior to telitacicept treatment, so the drop in scores after two weeks may be attributable to the effects of PLEX. Notedly, the patient has experienced herpes zoster infections following the combination of RTX and telitacicept, which may be associated with chronic adverse effects due to immunosuppression. To prevent infection, the patient’s general condition and immune status should be assessed at the time of administration of any immunosuppressive agent. Hence, the safety of this treatment strategy remains to be evaluated.

In refractory MuSK MG, RTX should be used as early as possible. As BLyS/APRIL targeted drugs, telitacicept not only inhibits the maturation and differentiation of B cells but reduces BAFF levels, and the combination may be an avenue to improve efficacy of RTX. Although combination therapy has shown potential short-term efficacy in our case, larger-scale clinical studies are needed to evaluate the impact of different treatment sequences on long-term efficacy and relapse rates. In addition, there is a great deal of variability in the response of MuSK MG patients to treatment, and treatment regimens should be tailored to each patient’s individual response.

## 6 Patient perspective

At the time of initial treatment, the patient was concerned about the side effects of RTX. After thorough communication, the patient agreed to use telitacicept, which was injected subcutaneously to make it convenient for her. The subsequent relapse made her anxious, and the patient eventually agreed to be treated with RTX in combination with telitacicept. Fortunately, her muscle strength, swallowing, and respiratory functions have finally improved. Her current daily activities are unrestricted, which eases the burden of care on her family, and she is satisfied with this combination therapy.

## Data Availability

The original contributions presented in the study are included in the article/**Supplementary Material**. Further inquiries can be directed to the corresponding authors.

## Supplementary material

Supplementary Figure 1 The serum samples were measured by Cytometric Bead Array. AChR= anti-acetylcholine receptor, LRP4= lipoprotein-related protein 4, MuSK= muscle-specific tyrosine kinase, RyR= ryanodine receptor, Titin=, VGCC= voltage-gated calcium channels.
