# Supplementary figures and images for: Telitacicept in combination with B-cell depletion therapy in MuSK antibody-positive myasthenia gravis: a case report and literature review
Source: Front Immunol. 2024 Nov 18;15:1456822. doi: 10.3389/fimmu.2024.1456822 (PMC11609065; doi:10.3389/fimmu.2024.1456822)

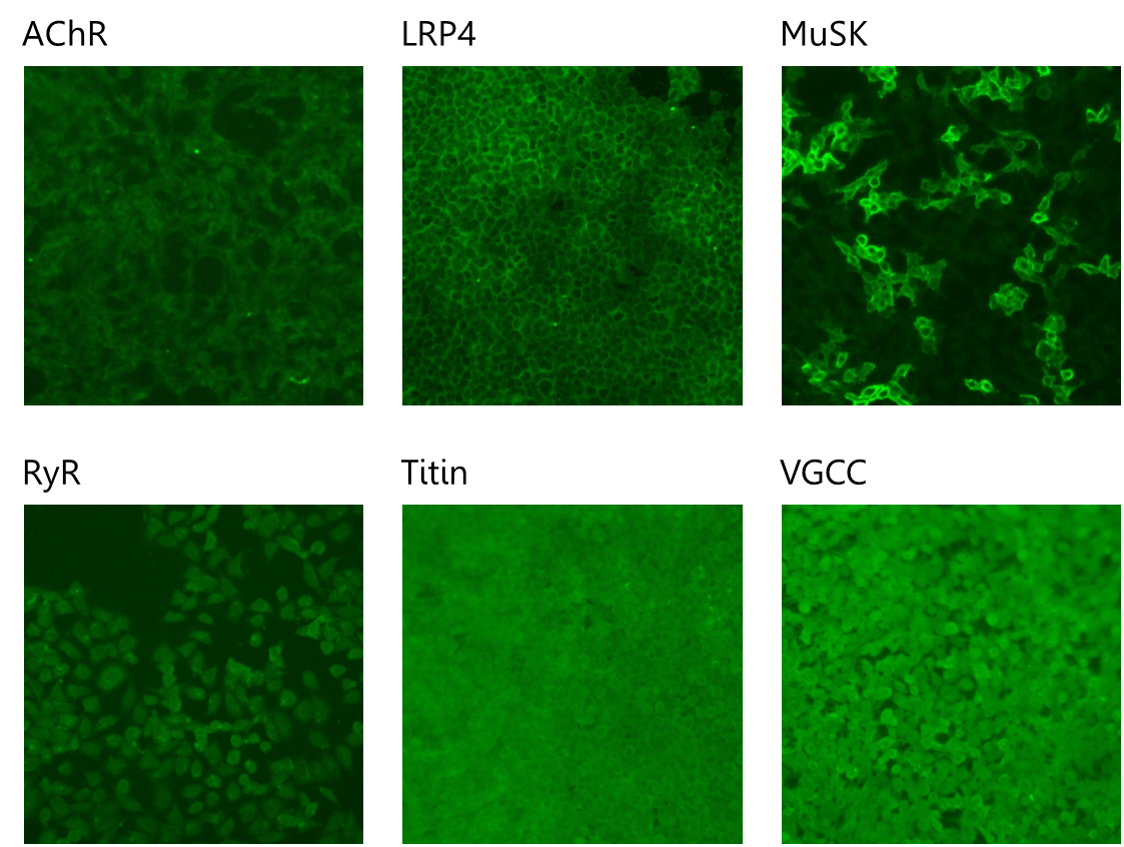

Supplement: Supplementary Figure 1 — The serum samples were measured by Cytometric Bead Array. AChR= anti-acetylcholine receptor, LRP4= lipoprotein-related protein 4, MuSK= muscle-specific tyrosine kinase, RyR= ryanodine receptor, Titin=, VGCC= voltage-gated calcium channels. [file Image1.tif]
